# Predicting the prognosis of patients with renal cell carcinoma based on the systemic immune inflammation index and prognostic nutritional index

**DOI:** 10.1038/s41598-024-76519-2

**Published:** 2024-10-23

**Authors:** Weiming Ma, Wei Liu, Yang Dong, Junjie Zhang, Lin Hao, Tian Xia, Xitao Wang, Conghui Han

**Affiliations:** 1 Suzhou Medical College of Soochow University, Suzhou, 215123 Jiangsu China; 2 Department of Urology, Xuzhou Central Hospital, Xuzhou, 221009 Jiangsu Provinve China; 3 Department of Medical oncology, Xuzhou Central Hospital, Xuzhou, 221009 Jiangsu China

**Keywords:** Renal cell carcinoma, Systemic immune inflammation index, Prognostic nutritional index, Risk factors, Nomogram, Cancer, Immunology, Nephrology, Risk factors, Urology

## Abstract

The aim of the study was to analyze and discuss the value of preoperative systemic immune inflammation index (SII) and prognostic nutritional index (PNI) in predicting the prognosis of patients with renal cell carcinoma (RCC) after operation, and to establish a nomogram prediction model for patients with RCC after operation based on SII and PNI. From January 2014 to December 2018, 210 patients with RCC who underwent surgical treatment at the Xuzhou Central Hospital were selected as the research object. The receiver operating characteristic curve (ROC) was used to determine the optimal cut-off value for preoperative SII, PNI, LMR, PLR, NLR and the patients were divided into groups according to the optimal cutoff values. The survival rate of patients was evaluated. The risk factors that affect the prognosis of patients with RCC were determined by LASSO and Cox regression analysis, and a prognostic nomogram was constructed based on this result. The bootstrap method was used for internal verification of the nomogram model. The prediction efficiency and discrimination of the nomogram model were evaluated by the calibration curve and index of concordance (C-index), respectively. The average overall survival (OS) of all patients was 75.385 months, and the 1-, 2-and 3-year survival rates were 95.5%, 86.6% and 77.2%, respectively. The survival curve showed that the 5-year OS rate of low SII group was significantly higher than that of high SII group (89.0% vs. 64.5%; *P* < 0.05), and low PNI group was significantly lower than those in high PNI group (43.4% vs. 87.9%; *p* < 0.05). There were significant differences between preoperative SII and CRP, NLR, PLR, LMR, postoperative recurrence, pathological type and AJCC stage (*P* < 0.05). There were significant differences between preoperative PNI and BMI, platelet, NLR, PLR, LMR, postoperative recurrence, surgical mode and Fuhrman grade (*P* < 0.05). The ROC curve analysis showed that the AUC of PNI (AUC = 0.736) was higher than that of other inflammatory indicators, followed by the AUC of SII (0.718), and the difference in AUC area between groups was statistically significant (*P* < 0.05). The results from multivariate Cox regression analysis showed that SII, PNI, tumor size, tumor necrosis, surgical mode, pathological type, CRP, AJCC stage and Fuhrman grade were independent risk factors for postoperative death of patients with RCC. According to the results of Cox regression analysis, a prediction model for the prognosis of RCC patients was established, and the C-index (0.918) showed that the model had good calibration and discrimination. The subject’s operating characteristic curve indicates that the nomogram has good prediction efficiency (the AUC = 0.953). Preoperative SII and PNI, tumor size, tumor necrosis, surgical mode, pathological type, CRP, AJCC stage and Fuhrman grade are closely related to the postoperative prognosis of patients with renal cell carcinoma. The nomogram model based on SII, PNI, tumor size, tumor necrosis, surgical mode, pathological type, CRP, AJCC stage and Fuhrman grade has good accuracy, discrimination and clinical prediction efficiency.

## Introduction

As one of the common malignant tumors in urinary system, renal cell carcinoma (RCC) accounts for 2% ~ 3% of all adult malignant tumors, among which renal clear cell carcinoma is one of the most common pathological types, accounting for 60% ~ 85% of renal cell carcinoma^1,2^. Due to RCC’s resistance to radiotherapy and chemotherapy, surgery remains the primary choice for early and locally advanced renal cell carcinoma^3^. However, due to individual differences, postoperative local recurrence (about 20-30%) and distant metastasis, the overall prognosis of patients with renal cell carcinoma is not ideal^4^. Therefore, it is particularly important to determine the risk factors of poor prognosis of patients with RCC in time and accurately, and to find biomarkers that can help clinicians make clinical decisions accurately, so as to improve the quality of life of patients and reduce the mortality^5^. At present, a large number of studies show that inflammatory reaction and malnutrition are closely related to the development and metastasis of malignant tumors and are important factors affecting the prognosis of patients with malignant tumors^6,7^. Systemic immune inflammation index (SII) was first proposed by Hu et al.^8^ in 2014. It is an inflammatory index that combines neutrophils, platelets and lymphocytes, and can reflect the degree of systemic inflammatory and immune state of the host. It has been proved to be an index for predicting the prognosis of patients with hepatocellular carcinoma, gastric cancer and lung cancer^8–10^. Prognostic Nutrition Index (PNI) was first proposed by Buzby et al.^11^ in 1980. It is a simple and easy nutritional index, which includes serum albumin level and lymphocyte count, and has been proved to be related to the prognosis of many cancer patients^12,13^. At present, some researchers combine SII and PNI and explore their correlation with the prognosis of cancer patients^14^, but there are few reports on the study of SII combined with PNI to predict the prognosis of patients with RCC after operation. This study aims to investigate the influence of preoperative SII and PNI levels on the prognosis of patients with RCC, and to construct a nomogram model of postoperative survival rate of RCC patients, so as to help clinicians provide better individualized treatment plans and follow-up strategies for patients with RCC.

## Materials and methods

### Data collection

From January 2014 to December 2018, 210 patients with RCC who underwent surgical treatment at the Xuzhou Central Hospital were selected as the research object. Surgical methods include radical nephrectomy and partial nephrectomy. Inclusion criteria: (1) RCC was diagnosed by pathology after operation; (2) No anti-tumor treatment before operation; (3) The patient’s medical records are complete and the postoperative follow-up is complete. Exclusion criteria: (1) Combined with blood or immune system diseases; (2) Combined with other tumors; (3) Acute or chronic inflammation before operation. This study was conducted according to the ethical principles of medical research involving human subjects in Helsinki Declaration. Patients and their families signed informed consent before operation.

### Analytical method

The general and clinical data of all patients were collected, including age, gender, diabetes, hypertension, body mass index (BMI), platelet, hemoglobin, tumor size, Ki-67, C- reactive protein (CRP), NLR, PLR, LMR, tumor necrosis, positive surgical margin, postoperative recurrence, surgical mode, pathological type, AJCC stage, Fuhrman grade, etc. Collect the blood sampling results of patients for the first time after admission, and the SII and PNI were calculated, where SII = platelet count (10^9^/L) × neutrophil count (10^9^/L) / lymphocyte count (10^9^/L); PNI = albumin level (g/L) + 5 × lymphocyte count (10^9^/L)^11^; NLR = neutrophil count (10^9^/L) / lymphocyte count (10^9^/L); PLR = platelet count (10^9^/L) / lymphocyte count (10^9^/L); LMR = lymphocyte count (10^9^/L) / monocyte count (10^9^/L)^15^. According to the optimal cut-off value, the patients were divided into high SII and low SII, high PNI and low PNI, high NLR and low NLR, high PLR and low PLR, high LMR and low LMR. Finally, the relationship between SII and PNI and the clinicopathological factors of the patients was analyzed.

### Follow-up

All postoperative patients were followed up regularly, including routine physical examination, chest X-ray, abdominal ultrasound, CT or MRI of urinary system and laboratory examination. Follow-up was conducted by outpatient service, SMS, telephone and WeChat. The follow-up plan is to follow up once every 3 months or 6 months for the first 2 years and once every year after 2 years. Overall survival (OS) is defined as the first day after operation to death or follow-up deadline, and the follow-up deadline is April 15, 2024 or the patient dies.

### Statistical analysis

SPSS 26.0 and R (4.4.0) software were used for statistical analysis. Receiver operating characteristic curve was used to determine the optimal cut-off value of SII, PNI, NLR, PLR, LMR, and patients were divided into low and high groups according to the values. The counting data were expressed by n (%), and the comparison between groups was made by χ^2^ test or Fisher test (*n* < 5). Kaplan-Meier method and log-rank method were used for survival analysis. Screening risk factors: LASSO analysis, which is especially suitable for reducing high-dimensional data, is used to select the risk factors that may affect the prognosis of patients with renal cell carcinoma as the best predictive features, and LASSO regression model is used to screen out features with non-zero coefficients^15^. For the factors selected in LASSO analysis, multivariate Cox regression analysis was used to verify the simplified prediction characteristics. The results were expressed by the values of risk ratio (HR), 95%CI and probability (P), and *P* < 0.05 was statistically significant. The screened independent risk factors were constructed by “rms” package in R software (version 4.4.0) and the corresponding nomogram prediction model was drawn. The Bootstrap method was used for internal verification of nomogram model. The index of concordance (C-index) is used to evaluate the discrimination of nomogram prediction model, and the test level is α = 0.05.

## Results

### Normal information

A total of 210 patients met the inclusion criteria, including 138 males (65.7%) and 72 females (34.3%), aged 33 ~ 82 years old, with an average of (58.1 ± 10.2) years. All 210 patients were diagnosed as RCC, and all of them were successfully discharged after surgery. By April 15, 2024, all 210 patients were followed up with a median follow-up time of 53 months. Of these, 39 patients died (18.6%) and 171 patients survived (81.4%). Postoperative recurrence was found in 16 cases (7.62%) and non-recurrence in 194 cases (92.38%).

### Determination of the best critical value of SII and PNI before operation

**Fig. 1:**
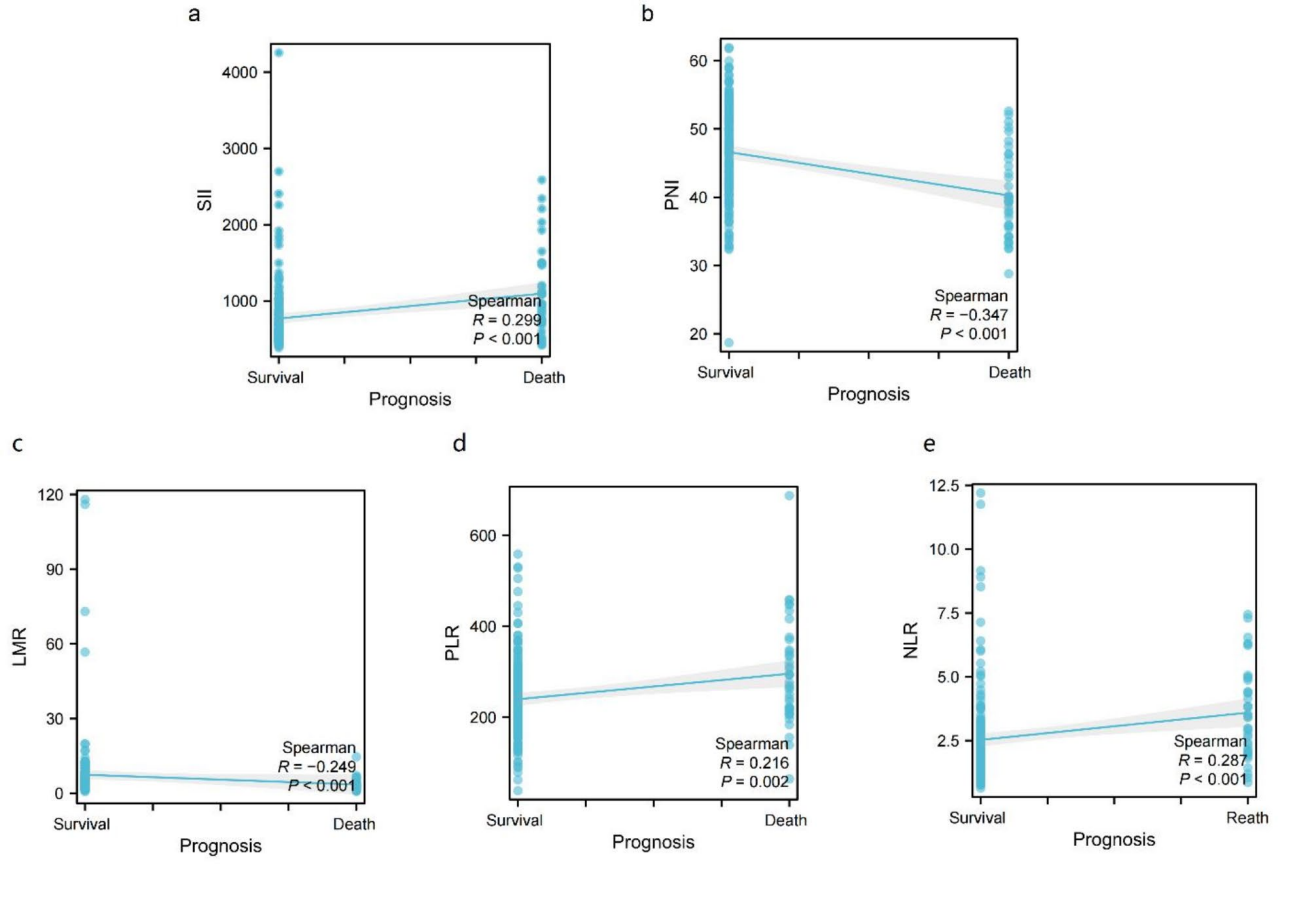
Correlation curve between SII, PNI, LMR, PLR and NLR and prognosis of patients with renal cell carcinoma. (**a**) SII, (**b**) PNI, (**c**) LMR, (**d**) PLR, (**e**) NLR.

**Fig. 2:**
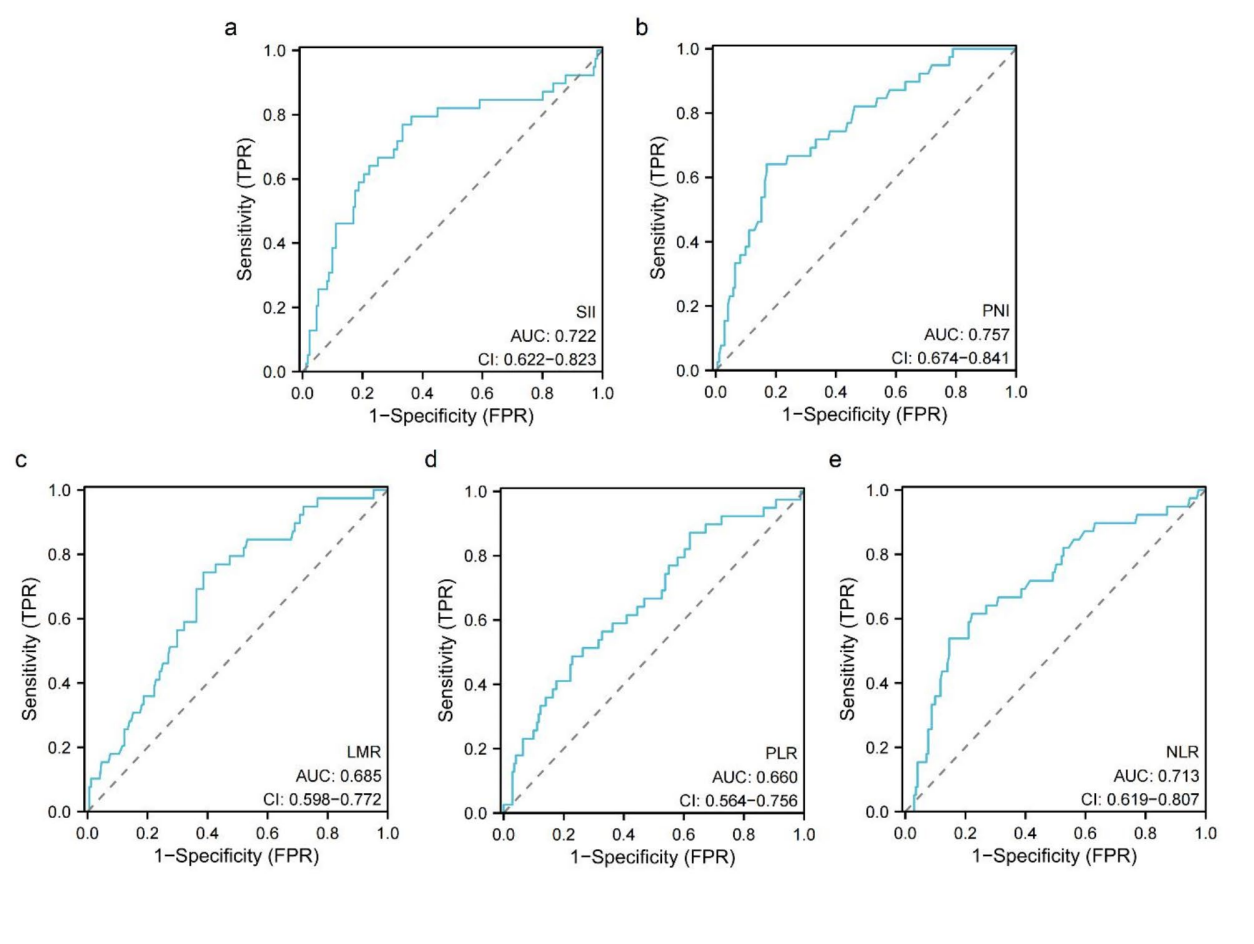
ROC curves of SII, PNI, LMR, PLR and NLR predicting the prognosis of patients with renal cell carcinoma. (**a**) SII, (**b**) PNI, (**c**) LMR, (**d**) PLR, (**e**) NLR.

Spearmen correlation analysis showed that SII was positively correlated with postoperative survival status of patients with renal cell carcinoma (*r* = 0.299, *P* < 0.001, Fig. 1a), PNI was negatively correlated with postoperative survival status of patients with renal cell carcinoma (*r*=-0.347, *P* < 0.001, Fig. 1b), LMR was negatively correlated with postoperative survival status of patients with renal cell carcinoma (*r*=-0.249, *P* < 0.001, Fig. 1c), PLR was positively correlated with postoperative survival status of patients with renal cell carcinoma (*r* = 0.216, *P* = 0.002, Fig. 1d), and NLR was positively correlated with postoperative survival status of patients with renal cell carcinoma (*r* = 0.287, *P* < 0.001, Fig. 1e), that is, the higher the preoperative values of SII, PLR and NLR, the lower the preoperative values of PNI and LMR, the higher the risk of death of patients. We determined the optimal cut-off values of SII, PNI, LMR, PLR and NLR by ROC analysis method, and took the value corresponding to the maximum Youden index as the optimal cut-off value, in which SII was 734.94, PNI was 40.23, LMR was 3.80, PLR was 290.67 and NLR was 2.90. According to the cut-off value, the patients were divided into two groups for further analysis: low SII group (SII ≤ 734.94, *n* = 123, Fig. 2a) and high SII group (SII > 734.94, *n* = 87, Fig. 2a); low PNI group (PNI ≤ 40.23, *n* = 54, Fig. 2b) and high PNI group (PNI > 40.23, *n* = 156, Fig. 2b); Low LMR group (LMR ≤ 3.80, *n* = 95, Fig. 2c) and high LMR group (LMR > 3.80, *n* = 115, Fig. 2c); Low PLR group (PLR ≤ 290.67, *n* = 152, Fig. 2d) and high PLR group (PLR > 290.67, *n* = 58, Fig. 2d); Low NLR group (NLR ≤ 2.90, *n* = 149, Fig. 2e) and high NLR group (NLR > 2.90, *n* = 61, Fig. 2e).

### Relationship between preoperative SII, PNI and clinicopathological factors in patients with RCC

There were significant differences between preoperative SII and CRP, NLR, PLR, LMR, postoperative recurrence, pathological type and AJCC stage (*P* < 0.05), but there was no significant differences between SII and age, gender, diabetes, hypertension, BMI, platelet, hemoglobin, tumor size, Ki-67, tumor necrosis, positive surgical margin, surgical mode and Fuhrman grade (*P* > 0.05). There were significant differences between preoperative PNI and BMI, platelet, NLR, PLR, LMR, postoperative recurrence, surgical mode and Fuhrman grade (*P* < 0.05), but there were no significant differences with age, gender, diabetes, hypertension, hemoglobin, tumor size, Ki-67, CRP, tumor necrosis, positive surgical margin, pathological type, and AJCC stage (*P* > 0.05), as shown in Table 1.

**Table 1:** Comparison of clinicopathological features of the two groups [n(%)]

Variables	Low SII group (*n* = 123)	High SII group (*n* = 87)	χ^2^	*P*	Low PNI group (*n* = 54)	High PNI group (*n* = 156)	χ^2^	*P*
Age (years)			0.419	0.518			0.831	0.362
≤ 60	68 (55.28)	52 (59.77)			28 (51.85)	92 (58.97)		
> 60	55 (44.72)	35 (40.23)			26 (48.15)	64 (41.03)		
Gender			0.060	0.807			3.364	0.067
Male	80 (65.04)	58 (66.67)			13 (24.07)	59 (37.82)		
Female	43 (34.96)	29 (33.33)			41 (75.93)	97 (62.18)		
Diabetes			0.003	0.959			0.703	0.402
None	83 (67.48)	59 (67.82)			39 (72.22)	103 (66.03)		
Yes	40 (32.52)	28 (32.18)			15 (27.78)	53 (33.97)		
Hypertension			0.000	0.9892			0.743	0.3892
None	92 (74.80)	65 (74.71)			38 (70.37)	119 (76.28)		
Yes	31 (25.20)	22 (25.29)			16 (29.63)	37 (23.72)		
BMI (kg/m^2^)			2.617	0.106			5.193	0.023
≤ 24	76 (61.79)	44 (50.57)			38 (70.37)	82 (52.56)		
> 24	47 (38.21)	43 (49.43)			16 (29.63)	74 (47.44)		
Platelet (×10^9^ /L)			0.542	0.462			5.411	0.020
≤ 300	50 (40.65)	31 (35.63)			28 (51.85)	53 (33.97)		
> 300	73 (59.35)	56 (64.37)			26 (48.15)	103 (66.03)		
Hemoglobin (g/L)			0.224	0.636			0.053	0.818
≤ 110	29 (23.58)	23 (26.44)			14 (25.93)	38 (24.36)		
> 110	94 (76.42)	64 (73.56)			40 (74.07)	118 (75.64)		
Tumor size (cm)			0.795	0.373			2.804	0.094
≤ 7	85 (69.11)	55 (63.22)			31 (57.41)	109 (69.87)		
> 7	38 (30.89)	32 (36.78)			23 (42.59)	47 (30.13)		
Ki-67			0.965	0.326			0.394	0.530
≤ 5%	104 (84.55)	69 (79.31)			46 (85.19)	127 (81.41)		
> 5%	19 (15.45)	18 (20.69)			8 (14.81)	29 (18.59)		
CRP (mg/L)			7.534	0.006			2.557	0.110
≤ 10	101 (82.11)	57 (65.52)			45 (83.33)	113 (72.44)		
> 10	22 (17.89)	30 (34.48)			9 (16.67)	43 (27.56)		
NLR			58.226	< 0.001			15.484	< 0.001
≤ 2.90	112 (91.06)	37 (42.53)			27 (50.00)	122 (78.21)		
> 2.90	11 (8.94)	50 (57.47)			27 (50.00)	34 (21.79)		
PLR			4.771	0.029			21.353	< 0.001
≤ 290.67	96 (78.05)	56 (64.37)			26 (48.15)	126 (80.77)		
> 290.67	27 (21.95)	31 (35.63)			28 (51.85)	30 (19.23)		
LMR			7.366	0.007			18.534	< 0.001
≤ 3.80	46 (37.40)	49 (56.32)			38 (70.37)	57 (36.54)		
> 3.80	77 (62.60)	38 (43.68)			16 (29.63)	99 (63.46)		
Tumor necrosis			0.002	0.966			0.168	0.682
None	93 (75.61)	66 (75.86)			42 (77.78)	117 (75.00)		
Yes	30 (24.39)	21 (34.14)			12 (22.22)	39 (25.00)		
Positive surgical margin			2.310	0.129			0.185	0.667
None	118 (95.93)	79 (90.80)			50 (92.59)	147 (94.23)		
Yes	5 (4.07)	8 (9.20)			4 (7.41)	9 (5.77)		
Postoperative recurrence			8.044	0.005			5.348	0.021
None	119 (96.75)	75 (86.21)			46 (85.19)	148 (94.87)		
Yes	4 (3.25)	12 (13.79)			8 (14.81)	8 (5.13)		
Surgical mode			1.012	0.314			13.241	< 0.001
Partial nephrectomy	75 (60.98)	47 (54.02)			20 (37.04)	102 (65.38)		
Radical nephrectomy	48 (39.02)	40 (45.98)			34 (62.96)	54 (34.62)		
Pathological type			4.367	0.037			2.392	0.122
Clear cell carcinoma	95 (77.24)	77 (88.51)			48 (88.89)	124 (79.49)		
Non-clear cell carcinoma	28 (22.76)	10 (11.49)			6 (11.11)	32 (20.51)		
AJCC stage			8.699	0.034			2.540	0.468
I stage	27 (21.95)	15 (17.24)			8 (14.81)	34 (21.79)		
II stage	66 (53.66)	34 (39.08)			25 (46.30)	75 (48.08)		
III stage	27 (21.95)	34 (39.08)			18 (33.33)	43 (27.56)		
IV stage	3 (2.44)	4 (4.60)			3 (5.56)	4 (2.57)		
Fuhrman grade			6.320	0.097			9.561	0.022
I stage	46 (37.40)	21 (24.15)			9 (16.67)	58 (37.18)		
II stage	62 (50.41)	48 (55.17)			34 (62.96)	76 (48.72)		
III stage	10 (8.13)	9 (10.34)			8 (14.81)	11 (7.05)		
IV stage	5 (4.06)	9 (10.34)			3 (5.56)	11 (7.05)		

### Relationship between SII and PNI and overall survival in patients with RCC

**Fig. 3:**
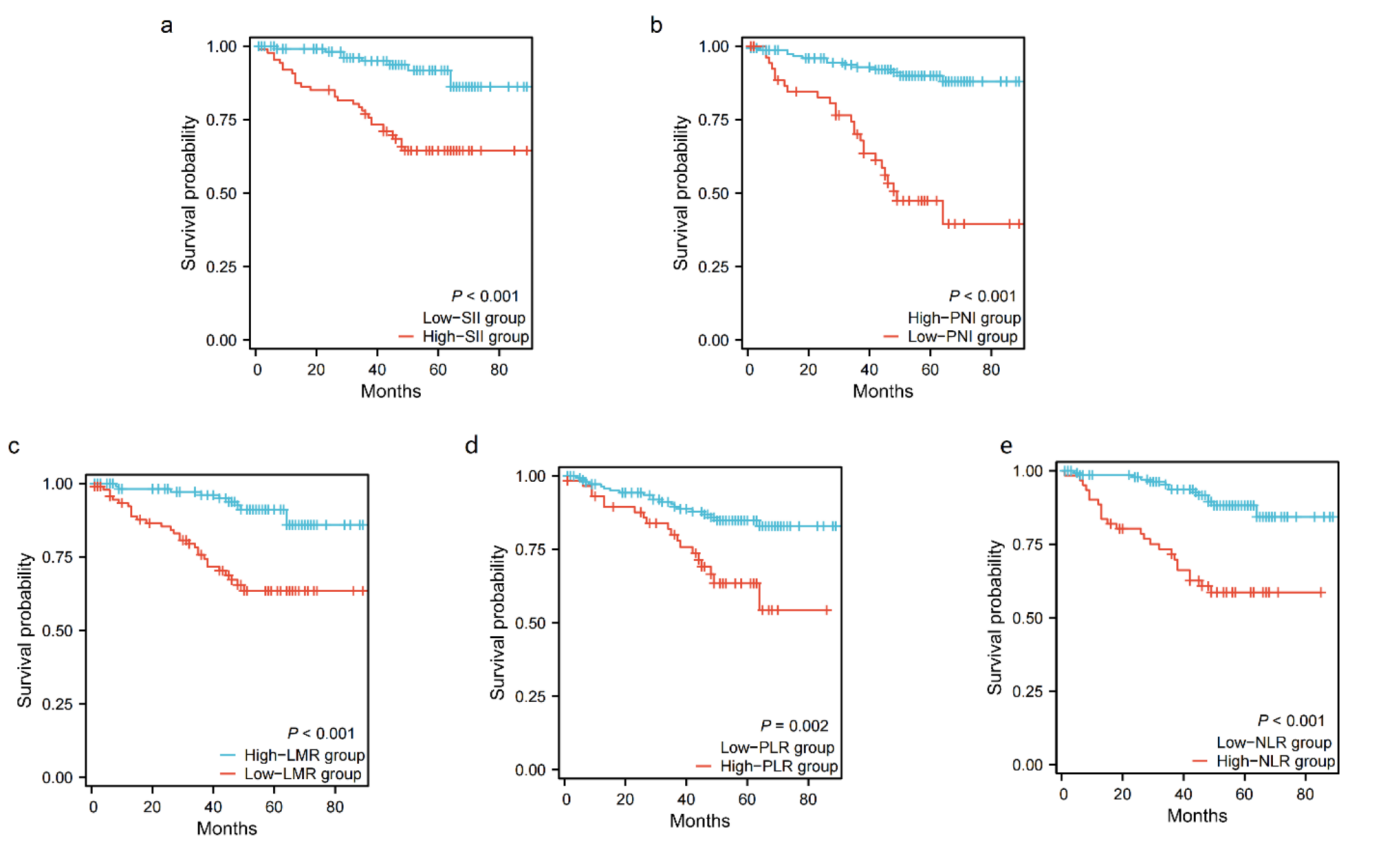
Overall survival curves of patients with renal cell carcinoma after surgery in the two groups. (**a**) SII, (**b**) PNI, (**c**) LMR, (**d**) PLR, (**e**) NLR.

The average survival time was 75.385 months (95% CI: 71.543–79.227), and the 5-year OS rate in the low SII group was significantly higher than that in the high SII group (89.0% vs. 64.5%; *p* < 0.001; Fig. 3a); The 5-year OS rate in low PNI group was significantly lower than that in the high PNI group (43.4% vs. 87.9%; *p* < 0.001; Fig. 3b); The 5-year OS rate in low LMR group was significantly lower than that in high LMR group (63.4% vs. 89.8%; *p* < 0.001; Fig. 3c); The 5-year OS rate in low PLR group was significantly higher than that in high PLR group (82.8% vs. 54.3%; *p* = 0.002; Fig. 3d); The 5-year OS rate in low NLR group was significantly higher than that in high NLR group (86.2% vs. 58.6%; *p* < 0.001; Fig. 3e).

### Comparison of prognostic value of some inflammatory indicators in patients with renal cell carcinoma after operation.

**Fig. 4:**
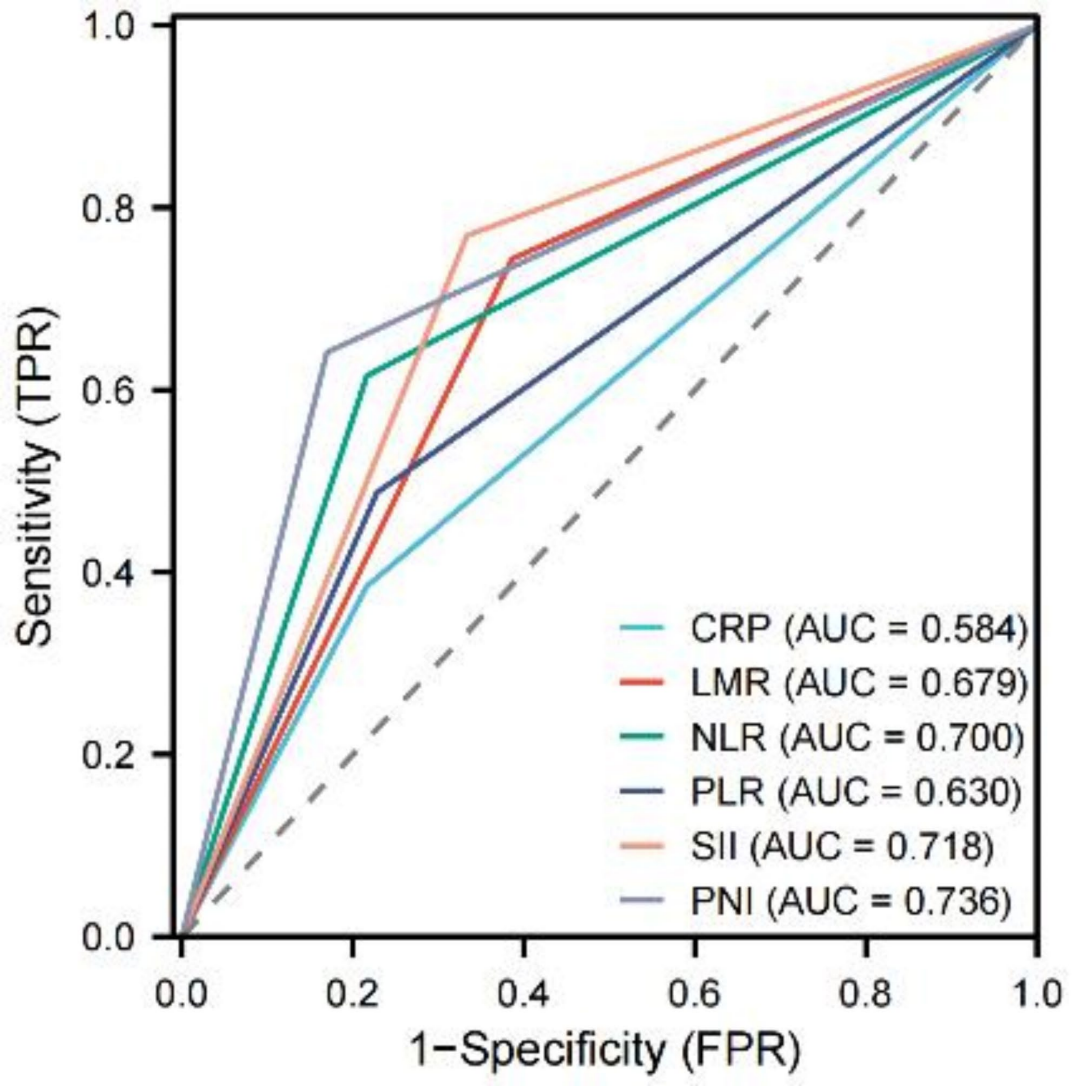
Comparison of SII, PNI and other inflammatory indicators in predicting the prognosis of patients with renal cell carcinoma.

According to the analysis of ROC curve, the AUC of SII = 0.718, PNI = 0.736, CRP = 0.584, LMR = 0.679, PLR = 0.630 and NLR = 0.700. The AUC of PNI is higher than other inflammatory indicators, followed by the AUC of SII, and the difference in AUC area between groups is statistically significant (*P* < 0.05). As new prognostic indicators, SII and PNI are expected to become prognostic indicators for patients with renal cell carcinoma, as shown in Fig. 4.

### The risk factors affecting overall survival in patients with RCC

**Fig. 5:**
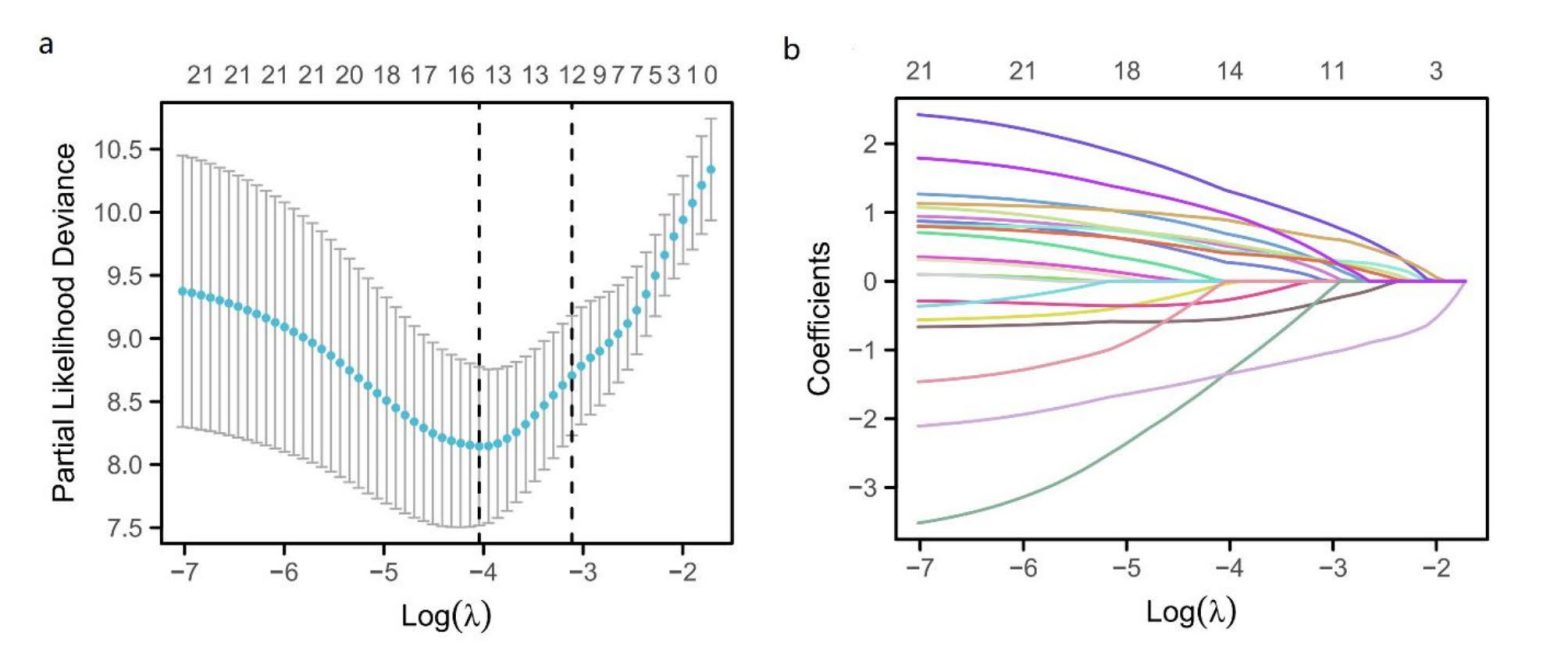
Screening prognostic risk factors of patients with renal cell carcinoma by LASSO analysis. (**a**) The relation curve between partial likelihood deviance and log (λ) is drawn. (**b**) LASSO coefficient distribution of 21 characteristic factors, in which the best λ produces 14 features with non-zero coefficients.

Based on 210 patients’ age, gender, diabetes, hypertension, BMI, platelet, hemoglobin, tumor size, Ki-67, CRP, NLR, PLR, LMR, tumor necrosis, positive surgical margin, surgical mode, pathological type, AJCC stage, Fuhrman grade, SII and PNI, 9 independent risk factors were screened out from 21 factors by LASSO analysis and multivariate Cox regression analysis, which were SII, PNI, tumor size, tumor necrosis, surgical mode, pathological type, CRP, AJCC stage and Fuhrman grade, respectively, as shown in Fig. 5; Table 2.

**Table 2:** Multivariate Cox regression models affecting the survival of patients with renal cell carcinoma.

Variables	β value	HR (95%CI)	*P* value
Hemoglobin (≤ 110 vs. >110)	−0.383	0.682 (0.306 ~ 1.518)	0.348
Hypertension (none vs. yes)	0.348	1.416 (0.568 ~ 3.531)	0.455
Tumor size (≤ 7 vs. >7)	0.856	2.355 (1.073 ~ 5.169)	0.033
Ki-67 (≤ 5% vs. >5%)	0.622	1.862(0.694 ~ 5.001)	0.217
NLR (≤ 2.90 vs. >2.90)	0.495	1.640 (0.620 ~ 4.340)	0.319
LMR (≤ 3.80 vs. >3.80)	−0.752	0.471 (0.185 ~ 1.200)	0.115
Tumor necrosis (none vs. yes)	1.106	3.023 (1.372 ~ 6.659)	0.006
Surgical mode (partial nephrectomy vs. radical nephrectomy)	2.089	8.077 (3.168 ~ 20.594)	< 0.001
Pathological type (clear cell carcinoma vs. non-clear cell carcinoma)	2.971	29.504 (3.296 ~ 115.414)	0.001
SII(≤734.94 vs. >734.94)	1.225	3.403 (1.312 ~ 8.825)	0.012
PNI(≤40.23 vs. >40.23)	−1.837	0.159 (0.069 ~ 0.369)	< 0.001
CRP (≤ 10 vs. >10)	1.577	4.841 (2.092 ~ 11.205)	< 0.001
AJCC stage			0.001
I stage	-	1 (reference)	-
II stage	1.469	4.343 (0.865 ~ 21.802)	0.074
III stage	2.288	9.858 (1.822 ~ 53.337)	0.008
IV stage	4.568	96.386 (9.425 ~ 985.664)	< 0.001
Fuhrman grade			0.027
I stage	-	1 (reference)	-
II stage	1.183	3.263 (1.020 ~ 10.435)	0.046
III stage	2.274	9.716 (2.229 ~ 42.348)	0.002
IV stage	1.518	4.564 (0.814 ~ 25.585)	0.084

### Nomogram creation and verification

**Fig. 6:**
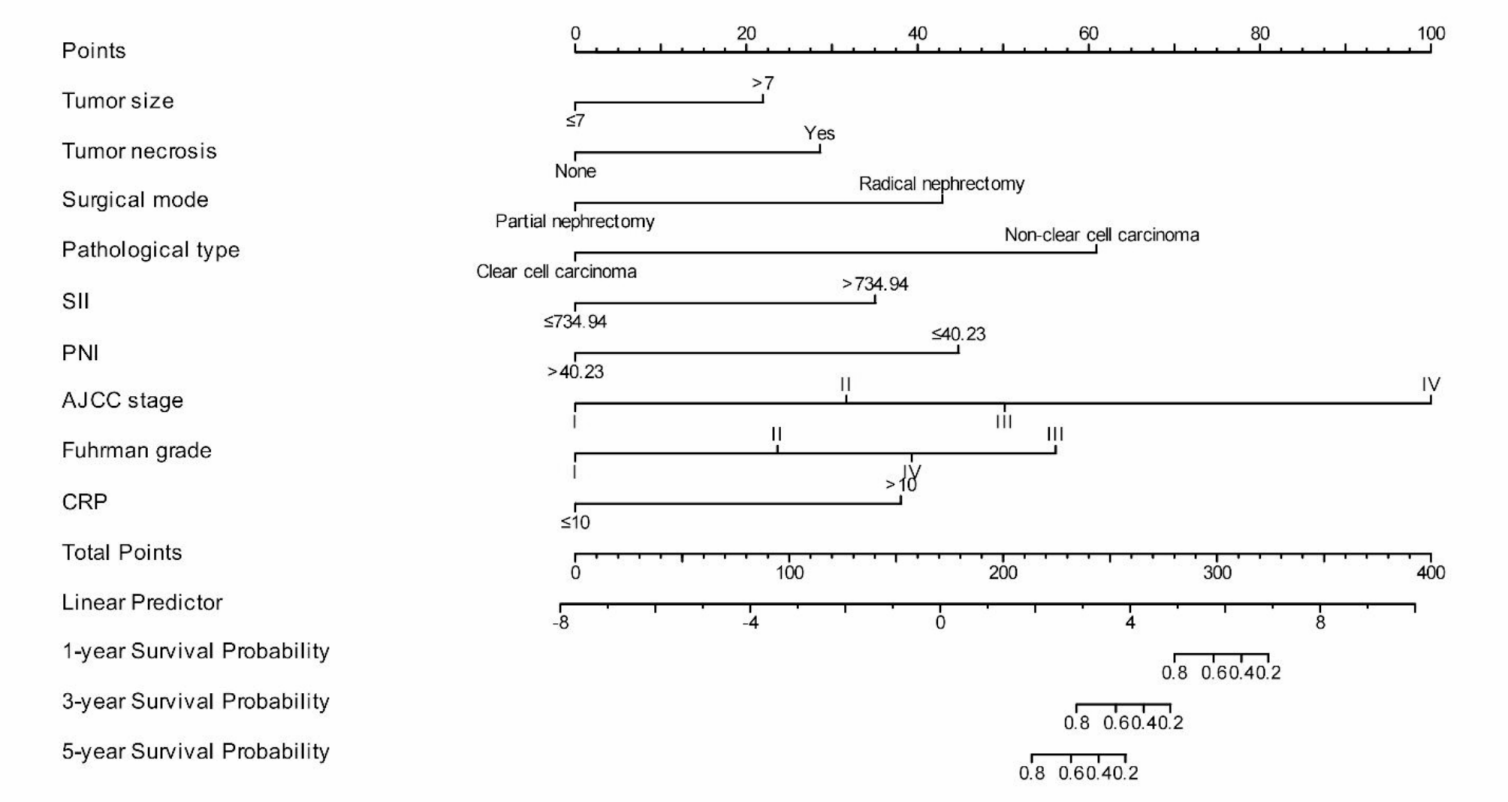
Nomogram prediction model for prognosis of patients with renal cell carcinoma.

**Fig. 7:**
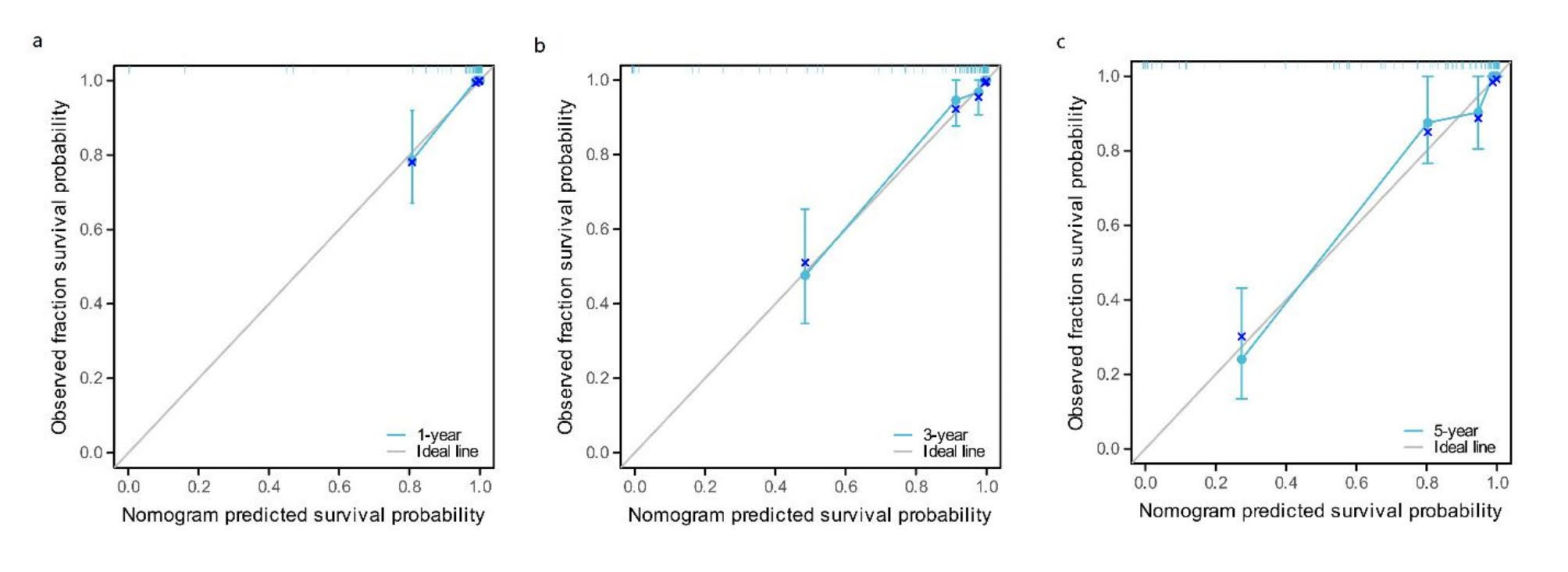
Calibration curve of the nomogram prediction model. (**a**) 1-year; (b) 3-year; (**c**) 5-year.

**Fig. 8:**
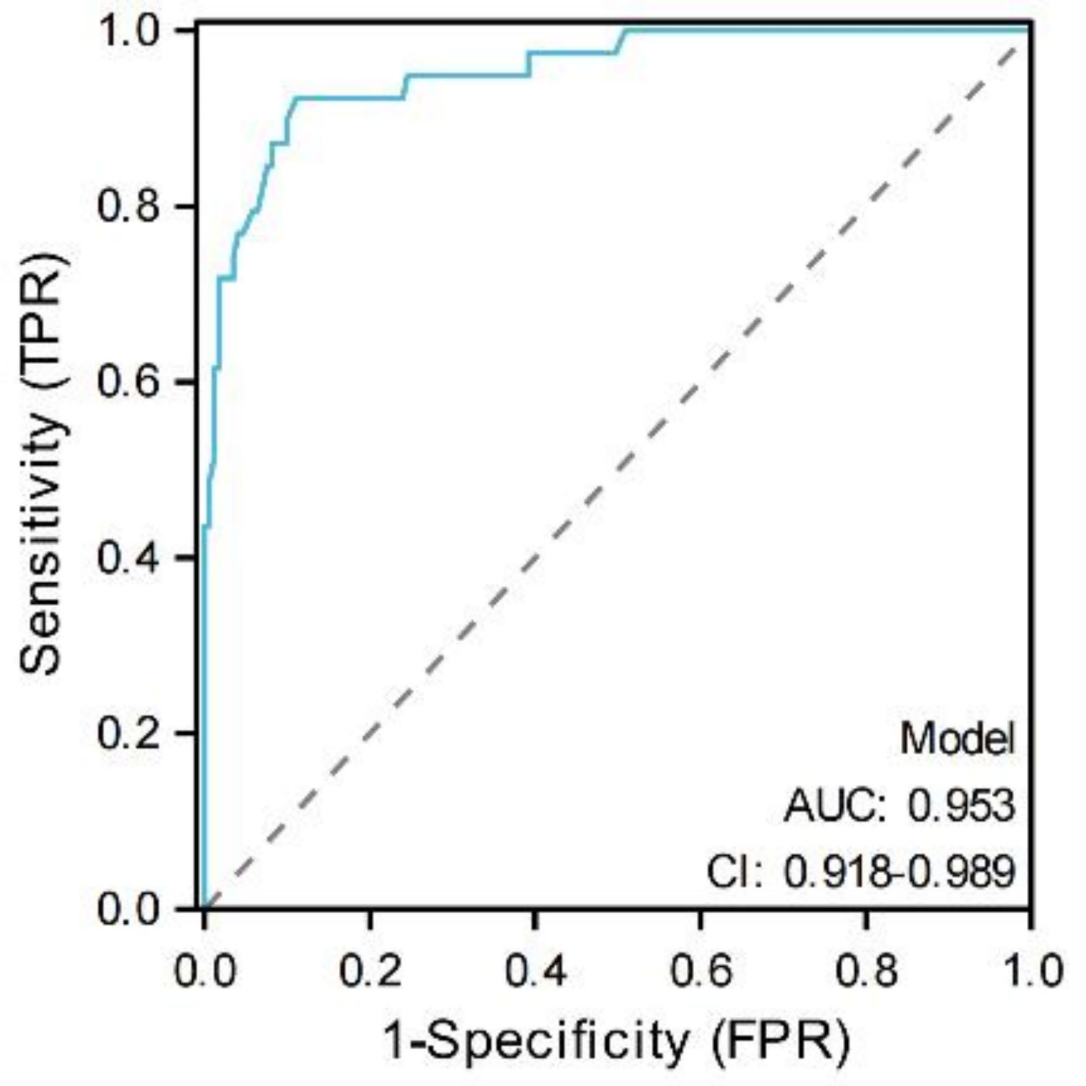
ROC curve of nomogram prediction model for predicting prognosis of patients with renal cell carcinoma.

Through multivariate Cox regression analysis, we found that SII, PNI, tumor size, tumor necrosis, surgical mode, pathological type, CRP, AJCC stage and Fuhrman grade were independent risk factors affecting the prognosis of patients with RCC (*P* < 0.05). The prediction model was constructed through the Cox regression formula based on the screened independent risk factors, and displayed it in the nomogram, as shown in Fig. 6. The C-index was 0.918 (0.897 ~ 0.939), indicating that the nomogram had good discrimination and consistency, and the calibration curve was very close to the ideal curve, indicating that the OS predicted by the nomogram model was consistent with the actual observations in the study cohort, as shown in Fig. 7. The ROC curve of the nomogram shows that the AUC of the predicted nomogram is 0.953 (95%CI: 0.918 ~ 0.989), which indicates that the nomogram has high prediction value (Fig. 8).

## Discussion

Renal cell carcinoma is a common urinary tumor. Although the early treatment effect is good, about 25% of patients still have local recurrence or distant metastasis after operation. Because RCC is not sensitive to radiotherapy and chemotherapy, the 5-year survival rate of patients with advanced renal cell carcinoma with recurrence or distant metastasis is extremely low^3^. Inflammation is closely related to multiple stages of tumor occurrence and development, and the value of SII and PNI in predicting the prognosis of various tumor patients has been confirmed^8–13^. However, there are few studies on the prognosis of patients with RCC. This study investigates the correlation between SII and PNI and the prognosis of patients with RCC, and constructs a nomogram prediction model to predict the prognosis of patients with RCC, so as to provide basis for clinical decision-making.

In this study, we found that SII was positively correlated with the postoperative survival status of patients with renal cell carcinoma (*r* = 0.299, *P* < 0.001), and PNI was negatively correlated with the postoperative survival status of patients with renal cell carcinoma (*r*=-0.347, *P* < 0.001), that is, the higher the preoperative SII value and the lower the PNI value, the higher the risk of death. In addition, we determined the optimal cut-off values of SII and PNI by ROC curve, and the cut-off values were 734.94 and 40.23 respectively. According to this value, 210 cases were divided into the low SII group and the high SII group, the low PNI group and the high PNI group. By analyzing the relationship between SII and the clinical characteristics of patients, we found that SII was significantly related to CRP, NLR, PLR, LMR, postoperative recurrence, pathological types and AJCC stage. On the one hand, SII is an inflammatory index based on neutrophils, platelets and lymphocytes, which can reflect the degree of inflammation and immune state of the body to a certain extent, while CRP, NLR, PLR and LMR are also widely used to reflect the inflammatory state of the body^14^, and there is a certain correlation between them. On the other hand, patients with poor pathological types and high AJCC stage reflect their poor prognosis to some extent, while poor immune status and their own inflammatory reaction can further worsen the prognosis of patients with RCC. In addition, by analyzing the relationship between PNI and the clinical characteristics of patients, we found that PNI was significantly related to BMI, platelet, NLR, PLR, LMR, postoperative recurrence, operation mode and Fuhrman grade (*P* < 0.05). On the one hand, PNI is an index based on albumin and lymphocytes, which can reflect not only the immune state of the body, but also its own nutritional state to some extent. And BMI is currently recognized as a commonly used indicator to reflect the body’s own nutritional status^16^, and platelet, NLR, PLR and LMR are also widely used to reflect the body’s inflammatory status^14^; On the other hand, the related literature has confirmed that surgical methods and Fuhrman grading are significantly related to the prognosis of patients with renal cell carcinoma^17,18^, and PNI is one of the important indicators to evaluate the nutritional status of patients. This study has confirmed that PNI is one of the key factors affecting the prognosis of patients with renal cell carcinoma. Finally, we found that preoperative SII and PNI were significantly correlated with postoperative recurrence of renal cell carcinoma (*P* < 0.05), that is, 12 cases (13.79%) in high SII group were higher than those in low SII group (4 cases, 3.25%), while 8 cases (14.81%) in low PNI group were higher than those in high PNI group (8 cases, 5.13%). It shows that SII and PNI can predict the postoperative recurrence of renal cell carcinoma to some extent. Of the 210 patients in this study, 194 patients (92.38%) did not relapse after operation, and none of them received radiotherapy or chemotherapy. Of the 16 cases (7.62%) who recurred after operation, 6 cases received chemotherapy (cisplatin + gemcitabine + tislelizumab), and 3 cases showed drug resistance. Among these 3 cases, the preoperative SII was at a high level, while the postoperative PNI was at a low level in 2 cases, indicating that SII and PNI may be related to the drug resistance of patients with renal cell carcinoma after operation. Considering the poor autoimmune state and malnutrition of the host, the internal environment of the body is obviously destroyed, thus obviously reducing the antiviral ability of the host and increasing the drug resistance of cancer cells^19^.

Nøst et al.^20^ believe that the markers of systemic inflammation are closely related to the increased risk and mortality of malignant tumors. Among them, neutrophils can inhibit the activation of T lymphocytes, help tumor cells escape immune surveillance, and enhance the invasion, metastasis and proliferation of cancer cells. Platelets can promote tumor angiogenesis, help circulating tumor cells to transmit through blood, and prevent them from immune attack. Lymphocytes have the ability of immune recognition, and inhibit tumor proliferation and metastasis by inducing tumor cell apoptosis^21^. SII is a comprehensive marker calculated from neutrophil count, platelet count and lymphocyte count, which can be used to evaluate the balance between systemic inflammation and immune response^22^. Studies have confirmed that SII is highly effective in predicting the prognosis of patients with solid tumors. In patients with hepatocellular carcinoma, gastric cancer and lung cancer, higher SII may promote tumor proliferation, progress and metastasis^8–10^, but there are few reports on the relationship between SII and RCC. LASSO analysis and multivariate Cox regression analysis showed that preoperative high SII was an independent risk factor for the prognosis of patients with RCC (*P* < 0.05). The results of this study are consistent with those of Steven Yang et al.^23^, indicating that SII is of high value in evaluating the prognosis of patients with RCC. The higher the preoperative SII, the worse the prognosis of patients.

Related research shows that lymphocyte and serum albumin levels are independent factors affecting the prognosis of patients with malignant tumors^24,25^. Serum albumin not only maintains plasma osmotic pressure and body nutrition, but also plays an important role in regulating body fluid distribution and acid-base balance. In addition, albumin, as the main component of plasma protein, is an important biomarker to predict the nutritional status of the body, which is considered to be related to the comorbidity and prognosis of some cancers^24^. Lymphocyte is an important part of cellular immunity, which inhibits the proliferation and invasion of tumor cells through cytokine-mediated cytotoxicity. However, the decrease of lymphocyte count or function will lead to tumor cells escaping from host immune surveillance, thus worsening the condition of tumor patients^26^. PNI is an index calculated according to serum albumin level and lymphocyte count^11^, which can be used to evaluate the immune and nutritional status of tumor patients. The decrease of lymphocyte count or serum albumin level will lead to the decrease of PNI. The results of this study showed that low preoperative PNI was an independent risk factor affecting the prognosis of patients with RCC (*P* < 0.05), suggesting that the lower preoperative PNI, the worse the prognosis of patients. Considering the reasons, it may provide favorable conditions for the proliferation and differentiation of tumor cells, promote the progress of malignant tumors, and thus affect the prognosis of patients with RCC. The results of this study are consistent with those of Ren et al.^27^. In addition, the results of this study also showed that CRP, tumor size, tumor necrosis, pathological type, surgical modes, AJCC stage and Fuhrman grade were also risk factors for postoperative death of patients with renal cell carcinoma. Among them, CRP was one of the important indicators to reflect the body’s own inflammatory state. Xia et al.^28^ retrospectively analyzed the clinical data of 985 patients with non-metastatic clear cell renal cell carcinoma (ccRCC), and found that CRP was independent prognostic factors of overall survival (OS) and metastasis-free survival (MFS) of ccRCC patients, and incorporating CRP into the traditional TNM staging system could improve its predictive performance. Tumor size is also a key factor affecting the prognosis of patients. It is reported that every 1 cm increase in the size of primary tumor in patients with renal cell carcinoma means an increase of 3.8% in the risk of death^29^. Zhang et al.^30^ found that tumor necrosis is related to CSS, OS, RFS and PFS in RCC patients, and can be used as a predictor of poor prognosis in RCC patients. Related literature has reported that pathological types are significantly related to the prognosis of patients with renal cell carcinoma, and the prognosis of patients with renal clear cell carcinoma is often better than that of patients with renal non-clear cell carcinoma^31^, which is consistent with the results of this study. In addition, AJCC stage and Fuhrman grade are recognized as important indicators to evaluate the prognosis of patients with renal cell carcinoma^32,33^, and the results of this study further confirm the correlation between AJCC stage and Fuhrman grade and the prognosis of patients with renal cell carcinoma. As for surgical modes, previous studies have confirmed that they are the key factors affecting the prognosis of patients, and clinicians can improve the prognosis of patients by optimizing surgical methods^34^. In addition, through ROC curve analysis, we found that the AUC of PNI (AUC = 0.736) was higher than that of other inflammatory indicators, followed by that of SII (0.718), and the difference in AUC area between groups was statistically significant (*P* < 0.05), indicating that SII and PNI, as new prognostic indicators, are expected to be tools for evaluating the prognosis of patients with renal cell carcinoma.

Finally, based on the independent risk factors (SII, PNI, tumor size, tumor necrosis, surgical mode, pathological type, CRP, AJCC stage and Fuhrman grade) affecting the prognosis of patients with RCC, a nomogram prediction model was constructed and verified. The verification results show that the nomogram model has good calibration, discrimination and prediction efficiency. The ROC curve of the nomogram shows that the area under the curve of the nomogram is 0.953, which is higher than that of SII (0.718) and PNI (0.736), indicating that the nomogram has high predictive value and can be effectively applied to predict the prognosis of patients with renal cell carcinoma.

There are some limitations in this study. On the one hand, this is a retrospective study of a small sample, and there may be selection bias in the process of data collection. On the other hand, this study only verified the research results internally, but not externally. Therefore, in the follow-up research, we should carry out more large-scale and multi-center research in order to improve the accuracy of the model.

In conclusion, preoperative SII, PNI, tumor size, tumor necrosis, surgical mode, pathological type, CRP, AJCC stage and Fuhrman grade are closely related to the postoperative prognosis of patients with RCC. The nomogram model based on the above indexes has good accuracy, discrimination and clinical application value.

## Data Availability

The datasets used and/or analyzed during the current study are available from the corresponding author on reasonable request.

## References

[1] Zhu, D. et al. A case of ipsilateral renal cell carcinoma complicated with clear cell and papillary renal cell carcinoma. *Int. J. Urol.*** 36** (4), 601–602. 10.3760/cma.j.issn.1673-4416.2016.04.039 (2016).

[2] China Medical Association Urology Health Promotion Branch, Urology Professional Committee of China Research Hospital Society. Consensus on the Safety of Partial Nephrectomy. *J. Mod. Urol. ***25**(6), 474–481500. 10.3969/j.issn.1009-8291.2020.06.003 (2020).

[3] Ljungberg, B. et al. European Association of Urology Guidelines on Renal Cell Carcinoma: The 2019 Update. *Eur. Urol.*** 75**(5), 799–810. 10.1016/j.eururo.2019.02.011 (2019). 30803729 10.1016/j.eururo.2019.02.011

[4] Jamil, M. L. et al. Long-term risk of recurrence in surgically treated renal cell carcinoma: A post hoc analysis of the Eastern Cooperative Oncology Group-American College of Radiology Imaging Network E2805 Trial Cohort. *Eur. Urol.*** 77**(2), 277–281. 10.1016/j.eururo.2019.10.028 (2020). 31703971 10.1016/j.eururo.2019.10.028

[5] Chen Pengliang, C. et al. microRNA and the invasion and metastasis of renal cell carcinoma. *Guangdong Med.*** 37**(19), 2978–2981. 10.3969/j.issn.1001-9448.2016.19.045 (2016).

[6] Sharma, B. R. & Kanneganti, T. D. NLRP3 inflammasome in cancer and metabolic diseases. *Nat. Immunol.*** 22** (5), 550–559. 10.1038/s41590-021-00886-5 (2021). 33707781 10.1038/s41590-021-00886-5 PMC8132572

[7] Simon, S. R. et al. Malnutrition screening in head and neck cancer patients with oropharyngeal dysphagia. *Clin. Nutr. ESPEN*. **44**, 348–355. 10.1016/j.clnesp.2021.05.019 (2021). 34330489 10.1016/j.clnesp.2021.05.019

[8] Hu, B. et al. Systemic immune-inflammation index predicts prognosis of patients after curative resection for hepatocellular carcinoma. *Clin. Cancer Res.*** 20** (23), 6212–6222. 10.1158/1078-0432.CCR-14-0442 (2014). 25271081 10.1158/1078-0432.CCR-14-0442

[9] Cao, X., Xue, J., Yang, H., Han, X. & Zu, G. Association of Clinical Parameters and prognosis with the pretreatment systemic Immune-inflammation index (SII) in patients with gastric Cancer. *J. Coll. Physicians Surg. Pak*. **31**(1), 83–88. 10.29271/jcpsp.2021.01.83 (2021). 33546540 10.29271/jcpsp.2021.01.83

[10] Liu, J. et al. Systemic immune-inflammation index, neutrophil-to-lymphocyte ratio, platelet-to-lymphocyte ratio can predict clinical outcomes in patients with metastatic non-small-cell lung cancer treated with nivolumab. *J. Clin. Lab. Anal.*** 33**(8), e22964. 10.1002/jcla.22964 (2019). 31282096 10.1002/jcla.22964 PMC6805305

[11] Buzby, G. P., Mullen, J. L., Matthews, D. C., Hobbs, C. L. & Rosato, E. F. Prognostic nutritional index in gastrointestinal surgery. *Am. J. Surg.*** 139**(1), 160–167. 10.1016/0002-9610(80)90246-9 (1980). 7350839 10.1016/0002-9610(80)90246-9

[12] Jiang, P., Li, X., Wang, S. & Liu, Y. Prognostic significance of PNI in patients with pancreatic Head Cancer undergoing laparoscopic pancreaticoduodenectomy. *Front. Surg.*** 9**, 897033. 10.3389/fsurg.2022.897033 (2022). 35722527 10.3389/fsurg.2022.897033 PMC9198448

[13] Nogueiro, J. et al. The impact of the prognostic nutritional index (PNI) in gastric cancer. *Langenbecks Arch. Surg.*** 407**(7), 2703–2714. 10.1007/s00423-022-02627-0 (2022). 35932298 10.1007/s00423-022-02627-0

[14] Ding, P. et al. Combined systemic immune-inflammatory index (SII) and prognostic nutritional index (PNI) predicts chemotherapy response and prognosis in locally advanced gastric cancer patients receiving neoadjuvant chemotherapy with PD-1 antibody sintilimab and XELOX: A prospective study. *BMC Gastroenterol.*** 22**(1), 121. 10.1186/s12876-022-02199-9 (2022). 35287591 10.1186/s12876-022-02199-9 PMC8919583

[15] Kidd, A. C. et al. Survival prediction in mesothelioma using a scalable Lasso regression model: Instructions for use and initial performance using clinical predictors. *BMJ Open. Respir Res.*** 5**(1), e000240. 10.1136/bmjresp-2017-000240 (2018). 29468073 10.1136/bmjresp-2017-000240 PMC5812388

[16] Venkatesh, N., Martini, A., McQuade, J. L., Msaouel, P. & Hahn, A. W. Obesity and renal cell carcinoma: Biological mechanisms and perspectives. *Semin Cancer Biol. ***94**, 21–33. 10.1016/j.semcancer.2023.06.001 (2023). 37286114 10.1016/j.semcancer.2023.06.001 PMC10526958

[17] Hong, S. K. et al. Application of simplified Fuhrman grading system in clear-cell renal cell carcinoma. *BJU Int. ***107** (3), 409–415. 10.1111/j.1464-410X.2010.09561.x (2011). 20804473 10.1111/j.1464-410X.2010.09561.x

[18] Husain, F. Z., Badani, K. K., Sfakianos, J. P. & Mehrazin, R. Emerging surgical treatments for renal cell carcinoma. *Future Oncol.*** 12**(7), 921–929. 10.2217/fon.15.362 (2016). 26892144 10.2217/fon.15.362

[19] Zhang, Y. et al. Ageing microenvironment mediates lymphocyte carcinogenesis and lymphoma drug resistance: From mechanisms to clinical therapy (review). *Int. J. Oncol.*** 64** (6), 65. 10.3892/ijo.2024.5653 (2024). 38757347 10.3892/ijo.2024.5653 PMC11095602

[20] Nøst, T. H. et al. Systemic inflammation markers and cancer incidence in the UK Biobank. *Eur. J. Epidemiol.*** 36**(8), 841–848. 10.1007/s10654-021-00752-6 (2021). 34036468 10.1007/s10654-021-00752-6 PMC8416852

[21] Xia, Y. et al. Systemic Immune inflammation index (SII), system inflammation response index (SIRI) and risk of all-cause mortality and cardiovascular mortality: A 20-year follow-up cohort study of 42,875 US adults. *J. Clin. Med.*** 12** (3), 1128. 10.3390/jcm12031128 (2023). 36769776 10.3390/jcm12031128 PMC9918056

[22] Xie, R. et al. Association between SII and hepatic steatosis and liver fibrosis: A population-based study. *Front. Immunol.*** 13**, 925690. 10.3389/fimmu.2022.925690 (2022). 36189280 10.3389/fimmu.2022.925690 PMC9520084

[23] Steven Yang, L. et al. Evaluation of the prognostic value of preoperative peripheral blood inflammation index for renal cell carcinoma. *J. Mod. Urol.*** 26**(7), 587–591. 10.3969/j.issn.1009-8291.2021.07.012 (2021).

[24] Zhao, J. et al. Prognostic role of pretreatment blood lymphocyte count in patients with solid tumors: A systematic review and meta-analysis. *Cancer Cell. Int. ***20**, 15. 10.1186/s12935-020-1094-5 (2020). 31938023 10.1186/s12935-020-1094-5 PMC6954501

[25] Zeeshan, F., Madheswaran, T., Panneerselvam, J., Taliyan, R. & Kesharwani, P. Human serum albumin as multifunctional nanocarrier for cancer therapy. *J. Pharm. Sci.*** 110**(9), 3111–3117. 10.1016/j.xphs.2021.05.001 (2021). 33989679 10.1016/j.xphs.2021.05.001

[26] Rauf, Z. et al. Lymphocyte detection for cancer analysis using a novel fusion block based channel boosted CNN. *Sci. Rep.*** 13**(1), 14047. 10.1038/s41598-023-40581-z (2023). 37640739 10.1038/s41598-023-40581-z PMC10462751

[27] Ren, W. et al. Clinical significance of prognostic nutritional index (PNI)-monocyte-to-lymphocyte ratio (MLR)-platelet (PLT) score on postoperative outcomes in non-metastatic clear cell renal cell carcinoma. *BMC Surg. ***23**(1), 117. 10.1186/s12893-023-02001-x (2023). 37165423 10.1186/s12893-023-02001-x PMC10170679

[28] Xia, W. K. et al. Prognostic significance of lymphocyte-to-monocyte ratio and CRP in patients with nonmetastatic clear cell renal cell carcinoma: A retrospective multicenter analysis. *Onco Targets Ther. ***9**, 2759–2767. 10.2147/OTT.S101458 (2016). 27274272 10.2147/OTT.S101458 PMC4869664

[29] Jiang, W. et al. Impact of primary tumor size on prognosis in patients with metastatic renal cell carcinoma receiving cytoreductive nephrectomy: A population study of a Chinese Center and the US SEER database. *Technol. Cancer Res. Treat.*** 20**, 15330338211019507. 10.1177/15330338211019507 (2021). 34032149 10.1177/15330338211019507 PMC8155752

[30] Zhang, L. et al. Tumor necrosis as a prognostic variable for the clinical outcome in patients with renal cell carcinoma: A systematic review and meta-analysis. *BMC Cancer*. **18**(1), 870. 10.1186/s12885-018-4773-z (2018). 30176824 10.1186/s12885-018-4773-z PMC6122538

[31] Sun, Z. et al. Construction of a lactate-related prognostic signature for predicting prognosis, tumor microenvironment, and immune response in kidney renal clear cell carcinoma. *Front. Immunol. ***13**, 818984. 10.3389/fimmu.2022.818984 (2022). 35250999 10.3389/fimmu.2022.818984 PMC8892380

[32] Elkassem, A. A., Allen, B. C., Sharbidre, K. G., Rais-Bahrami, S. & Smith, A. D. Update on the role of imaging in clinical staging and restaging of renal cell Carcinoma based on the AJCC 8th Edition, from the AJR Special Series on Cancer Staging. *AJR Am. J. Roentgenol. ***217** (3), 541–555. 10.2214/AJR.21.25493 (2021). 33759558 10.2214/AJR.21.25493

[33] Zhang, Q. et al. Metabolic syndrome is an independent risk factor for Fuhrman Grade and TNM stage of renal clear cell carcinoma. *Int. J. Gen. Med.*** 15**, 143–150. 10.2147/IJGM.S346972 (2022). 35023952 10.2147/IJGM.S346972 PMC8743490

[34] Patel, H. D., Srivastava, A. & Editorial Optimizing surgical procedures in renal cancers to improve patient outcomes. *Front. Oncol.*** 12**, 1019946. 10.3389/fonc.2022.1019946 (2022). 36237338 10.3389/fonc.2022.1019946 PMC9552350

